# Fetal bone as a foreign body in the urinary bladder: a case report

**DOI:** 10.4076/1752-1947-3-8994

**Published:** 2009-08-27

**Authors:** Muhammad Akram Malik, Abdul Ghaffar Rehan, Iftikhar Ahmad, Tanveer Ahmad

**Affiliations:** 1 Department of Urology, Madina Teaching Hospital, Faisalabad, Pakistan; 2 Department of Surgery, Madina Teaching Hospital, Faisalabad, Pakistan

## Abstract

**Introduction:**

A wide range of objects have been inserted into the urinary bladder posing a challenge to urologists for diagnosis and management. Most cases are associated with psychiatric disorders, senility, intoxication or autoerotic stimulation. The frequency of such cases renders them important medical conditions of the genitourinary system.

**Case presentation:**

Our case is an unusual one since fetal bone as a foreign body in the urinary bladder has not been reported in the literature. During dilatation and curettage, fetal bone migrated into the bladder wall of a 37-year-old woman and was endoscopically removed 7 years later.

**Conclusion:**

A foreign body in the urinary bladder is rare and in most cases are self-inserted. Iatrogenic insertion is relatively rare especially during gynecological intervention. The presence of a foreign body in the bladder should be kept in mind when dealing with unusual cases of lower urinary tract symptoms.

## Introduction

Foreign bodies are common in the upper gastrointestinal and respiratory tract. However, foreign bodies in the urinary bladder are relatively rare. The variety of foreign bodies found inserted into or externally attached to the genitourinary tract is extremely varied and has included fish hooks, metal rods, hair pins, screws, pellets, wires, wooden sticks, pieces of fish and telephone cables [1]-[3]. Most cases are associated with psychiatric disorders, senility, intoxication or autoerotic stimulation [4]. Such cases are important additions to the medical conditions of the genitourinary system [5]. Most patients are usually too ashamed to admit to the introduction of a foreign body and usually present with dysuria, urinary frequency, hematuria, suprapubic pain, swelling of the penis and external genitalia, extravasations or abscess formation [6]. Diagnosis is based largely on history and clinical examination. However, radiological and cystoscopic studies are often required to confirm the diagnosis and to plan management [7]. The management includes extraction of the foreign body and prevention of long-term complications in addition to assessment of patient motivation. In this case report, we present a rare and probably the first report of this type of foreign body in the urinary bladder.

## Case presentation

**Figure 1:**
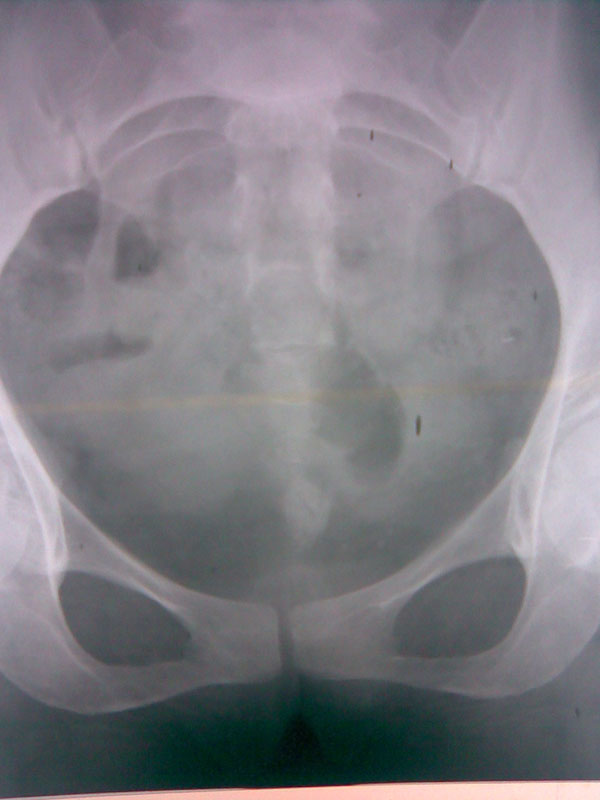
**X-ray of the kidney, ureter and bladder showing radio-opaque irregular shadow in the posterior wall of the bladder**.

**Figure 2:**
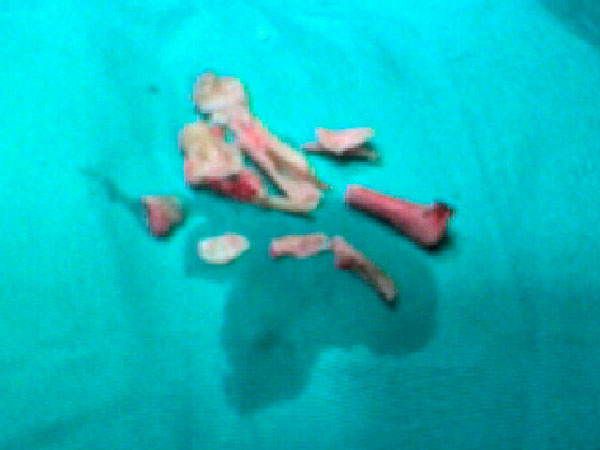
**Bony pieces after removal from the bladder**.

A 37-year-old woman presented to our outpatient department with hematuria, increased urinary frequency and suprapubic pain for the previous 7 years. Her menstrual history revealed irregularity with no conception during this period. She underwent dilatation and curettage 7 years previously for amenorrhea of 4 months' duration. Her gynecological and abdominal examinations were unremarkable. Ultrasonography of the kidney, ureter and bladder (KUB) showed normal upper urinary tracts and a 2 × 3 cm irregularly shaped stone at the trigone, which did not move with side-to-side movement of the patient. X-ray KUB showed an irregular radio-opaque object in the pelvis 2 cm above the symphysis pubis (Figure 1). On cystoscopy, a small hard object was found to be projecting inside the bladder at the level of the ureteric bar close to the left ureteric orifice. Part of the object was inside the bladder and the remainder embedded in the bladder wall. A gentle attempt with forceps and stone punch failed to retrieve the object. Resection of the mucosa by resectoscope had to be performed and pieces of the hard object were removed endoscopically. The object was palpable per vaginally during the procedure with intact mucosa. Macroscopically, the pieces looked like bony or wooden material with overlying calcium deposition (Figure 2). The patient was catheterized for 5 days. Postoperative recovery was uneventful and urinary symptoms were relieved after 2 weeks. Histopathology proved the objects to be pieces of bone showing trabeculae, fibrocollagenous tissues and inflammatory cells.

## Discussion

Every conceivable object has been inserted into the bladder presenting a challenge of diagnosis and management to the urologist [8]. The most suitable method of removing the foreign body depends upon the size and mobility of the object in the genitourinary tract [9]. Numerous cases of foreign bodies of unusual nature in the urethra and bladder have been reported in the literature [9]. Such foreign bodies were introduced for sexual stimulation or during intoxication.

Shame and humiliation may prevent these patients from volunteering the reason. Even when the objects are extracted and presented to the patients, the majority deny any knowledge. Various methods for removal of foreign bodies from the bladder have been described including cystoscopy, suprapubic cystostomy and injection of solvents. Endoscopic retrieval is the treatment of choice. Grasping instruments may be required including grasping forceps, stone basket, stone punch and other modified instruments [1,3,9].

In our patient, we suspected a foreign body in the urinary bladder on the basis of symptoms and radiological studies. This may be the first case where fetal bone has migrated into the bladder and was retained in the urinary bladder wall for 7 years, as no such case has been reported in the literature.

It is thought that injury to the bladder wall occurred during attempted abortion and bony pieces penetrated into the bladder. The perforation sealed itself on the vaginal side and the patient suffered symptoms due to the presence of the foreign body in the bladder wall.

The presence of bone in the urinary bladder and its removal have been reported by Garcia Rojo *et al.* in 1993 [10], however that object was self-introduced. Iatrogenic introduction of a foreign body into the bladder has been reported in the literature but this may be the first reported case of iatrogenic introduction of fetal bony parts [11]-[13]. Attempted abortion leading to bladder and vaginal perforation and the presence of fetal bones in the bladder for more than 7 years reflects the health care system in Pakistan and possibly other developing countries.

An unqualified "Dai" (birth attendant) is usually involved in deliveries, abortions, dilatation and curettage in remote areas of Pakistan. Perforation of the uterus, bladder, intestine and rectum is not an uncommon complication under these circumstances. Trained birth attendants, female health workers and female health visitors have been trained and appointed in rural areas of the country but much planning and work is still required to improve the obstetrical and gynecological services in these areas.

## Conclusions

Foreign bodies in the urinary bladder are rare. Radiological investigations are usually required for their management and endoscopic removal is the treatment of choice. Iatrogenic insertion reflects the deficiency of trained health care professional in remote areas of Pakistan. World organizations should help developing nations in the provision of better health care facilities.

## Abbreviation

KUB: kidney, ureter and bladder.

## Consent

Written informed consent was obtained from the patient for publication of this case report and any accompanying images. A copy of the written consent is available for review by the Editor-in-Chief of this journal.

## Competing interests

The authors declare that they have no competing interests.

## Authors' contributions

MAM contributed to the management of the patient and writing this case report manuscript. AGR contributed in revising the manuscript and gave final approval of the final version. IA contributed to the management of the patient and revising the manuscript. TA contributed to the management of the patient and revising the manuscript.
